# Different Doses of Methamphetamine Are Needed to Produce Locomotor or Blood Pressure Sensitization in Mice

**DOI:** 10.3390/life14060723

**Published:** 2024-06-03

**Authors:** Carla Letizia Busceti, Domenico Bucci, Massimiliano De Lucia, Michela Ferrucci, Mariarosaria Scioli, Albino Carrizzo, Ferdinando Nicoletti, Carmine Vecchione, Francesco Fornai

**Affiliations:** 1 Istituto di Ricovero e Cura a Carattere Scientifico (IRCCS) Neuromed, 86077 Pozzilli, Italy; domenico.bucci@neuromed.it (D.B.); massidelucia.m@libero.it (M.D.L.); stabulario@neuromed.it (M.S.); albino.carrizzo@gmail.com (A.C.); ferdinandonicoletti@hotmail.com (F.N.); cvecchione@unisa.it (C.V.); 2 Department of Translational Research and New Technologies in Medicine and Surgery, University of Pisa, 56126 Pisa, Italy; michela.ferrucci@unipi.it; 3 Department of Medicine, Surgery and Dentistry “Scuola Medica Salernitana”, University of Salerno, 84081 Baronissi, Italy; 4 Department of Physiology and Pharmacology, University Sapienza, 00185 Roma, Italy

**Keywords:** methamphetamine, hypertension, catecholamines, sensitization, mice

## Abstract

Methamphetamine (METH) exposure increases locomotor sensitization. However, no study has explored the occurrence of cardiovascular sensitization. The present study, carried out in mice, analyzed the following: (i) METH sensitization extending to systolic blood pressure (SBP); (ii) a potential correlation between ambulatory and cardiovascular sensitization; and (iii) morphological alterations within meso-striatal, meso-limbic and pontine catecholamine systems including c-fos expression. Locomotor activity, SBP and occurrence of morphological alterations of catecholaminergic neurons were assessed in *C57Bl/6J* mice following daily i.p. injections of either saline or METH (1, 2 or 5 mg/kg) for 5 consecutive days and following 6 days of withdrawal. Reiterated exposure to the lower doses of METH (1 mg/kg and 2 mg/kg) produced in mice locomotor sensitization without altering SBP. In contrast, repeated treatment with the highest dose of METH (5 mg/kg) produced sensitization of SBP in the absence of locomotor sensitization. No morphological alterations but increases in c-fos expression within neurons of locus coeruleus and nucleus accumbens were detected. The present data suggest that METH produces plastic changes that extend beyond the motor systems to alter autonomic regulation. This cardiovascular sensitization occurs independently of locomotor sensitization. The persistency of increased blood pressure may underlie specific mechanisms operating in producing hypertension.

## 1. Introduction

Methamphetamine (METH) is a widely abused drug in the Western world, which may lead to a number of deleterious effects, both when administered acutely or following repeated administrations [1,2,3]. Most studies exploring the neurobiology of METH are focused on the central nervous system (CNS), with a special emphasis on the basal ganglia [3,4]. In fact, it is well known that METH is a psychostimulant, which induces an increase in locomotor activity [5,6] and promotes nigrostriatal dopamine (DA) release and nigrostriatal dopamine toxicity in mice [7,8], rats [9] and primates [10,11]. All these effects are connected as psychostimulation and increases in locomotor activity are greatly sustained by DA release, which, in turn, is a major determinant of METH-induced neurotoxicity [3,12]. When exposure to METH is reiterated, behavioral and motor sensitization may occur [6], with increasing effects produced by the same dose of METH when administered a number of times. Methamphetamine-induced sensitization can be measured in mice by increased ambulatory activity following repeated dosing [6,13]. These effects have been extensively investigated both in the dorsal and ventral striatum, where most locomotor activity induced by METH is generated [14,15,16,17].

In fact, when METH administration is reiterated, both psychostimulation and locomotor stimulation are enhanced in the process of METH-induced sensitization. Reiterations of high doses of METH also produce severe DA toxicity expressed by a loss of integrity at the level of the nigrostriatal DA system. Apart from psychomotor stimulation and nigrostriatal toxicity, METH administration produces remarkable effects on the cardiovascular system, mainly expressed by a marked increase in blood pressure in mice [18], rats [19], sheep [20], monkeys [21] and humans [22]. In fact, following METH intake, cardiovascular events such as stroke are common in human patients [23,24,25], which may lead to fatal events [23,26,27]. The effects induced by a single dose of METH on blood pressure consist of a transient increase followed by a prolonged decrease of cerebral blood flow below control levels [18]. Although psychomotor sensitization following repeated exposure to METH is extensively characterized, no study so far has investigated whether a permanent increase in blood pressure may occur following reiterated METH exposure in mice and whether such an increase follows up a sensitization pattern similar to that of locomotor activity. When this is the case, it would be interesting to analyze whether the doses of METH needed to produce a persistent and sensitized increase in blood pressure correspond to the doses of METH that are required to produce behavioral sensitization or whether higher/lower doses are needed. Again, since repeated administration of high doses of METH may produce nigrostriatal toxicity, it is important to analyze whether the occurrence of sensitized blood pressure in response to METH is concomitant either to damage to the DA nigrostriatal system or an alteration of the mesolimbic DA system. Again, since METH activity profoundly modifies norepinephrine (NE)-containing neurons in the pontine nucleus locus coeruleus (LC) [28], which is deeply involved in blood pressure adaptive changes [29], a morphological analysis was extended to LC. In addition, immunohistochemistry for the immediate early gene *c-fos* was carried out to roughly assess whether activation of these areas was produced, including the rostral ventrolateral medulla RVLM, which overlaps with the A1/C1 area. In fact, this latter region is profoundly involved in regulating blood pressure [30], mainly through glutamatergic neurons projecting to LC [31]. This was carried out specifically following dosing of METH, which induced persistent changes in blood pressure (highest dose: 5 mg/Kg). In the present study, the occurrence of blood pressure sensitization was investigated along with the potential persistency of increases in blood pressure following repeated METH exposure. In the second part of this study, the altered morphology of DA neurons outsourcing the meso-striatal and meso-limbic systems was investigated. Again, in keeping with the role of LC in modulating METH activity and its prominent effects on controlling blood pressure, altered morphology was assessed within this nucleus. Finally, the occurrence of increased c-fos expression as a morphological marker of neuronal activation was carried out in these areas, including the A1/C1 region. The present study questioned whether METH-induced sensitization may extend to cardiovascular control, with marked implications for the onset of cardiovascular disorders.

## 2. Materials and Methods

### 2.1. Materials

METH hydrochloride was purchased from Sigma Aldrich (St. Louis, MO, USA).

### 2.2. Animals

*C57Bl/6J* mice were originally provided by Charles River (Charles River, Calco, LC, Italy) and bred at IRCCS Neuromed according to the FELASA recommendations for the health monitoring of mouse colonies housed in conventionally controlled animal facilities.

Two-month-old *C57Bl/6J* male mice were used for this study. Only male mice were used for experiments to avoid any potential interference of ovarian steroids on METH-induced neuronal plasticity and cerebrovascular function.

Mice were housed under controlled conditions (temperature, 22 °C; humidity, 40%) with a 12 h light/dark cycle and food and water ad libitum. This study was approved by the Italian Ministry of Health (authorization #1065/2016-PR). All efforts were made to minimize animal suffering and reduce the number of animals used.

### 2.3. Experimental Strategy

**Figure 1:**
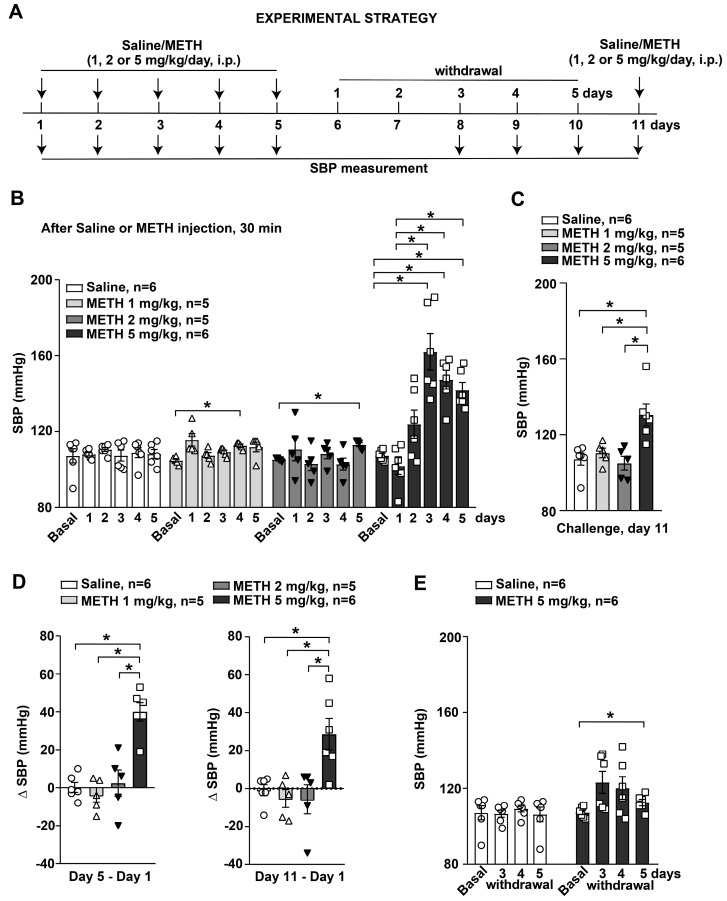
Reiterated exposure to methamphetamine (METH) induces systolic blood pressure (SBP) sensitization in mice. (**A**) Diagram showing the experimental strategy used for monitoring SBP values in mice subjected to repeated exposure to saline or METH. Mice were daily intraperitoneally (i.p.) injected with saline orMETH(1, 2 or 5 mg/kg) for 5 days and then re-challenged with saline or METH following 6 days of withdrawal at day 11. SBP was monitored by the tail-cuff method before treatments (basal) 30 min after each injection with saline or METH and at 3–5 days of withdrawal following the repeated injections. (**B**) SBP values monitored in mice treated with saline or METH (1, 2 or 5 mg/kg, i.p.) for 5 days. Values are reported as the means +/− S.E.M. * *p* < 0.05 (one-way ANOVA for repeated measures with Tukey’s test for multiple comparisons). * *p* < 0.05 vs. basal (before treatment) or day 1 (30 min after the first injection) values. (**C**) SBP values monitored in mice 30 min after the challenge with saline or METH performed following 6 days of withdrawal on day 11. Values are the means +/− S.E.M. * *p* < 0.05 (one-way ANOVA with Tukey’s test for multiple comparisons) vs. saline, METH (1 mg/kg) or METH (2 mg/kg) values. The difference in SBP values (Δ SBP) monitored at 30 min after saline or METH injections between day 5 and day 1 or day 11 and day 1 is shown in (**D**). Values are the means +/− S.E.M. * *p* < 0.05 (one-way ANOVA with Tukey’s test for multiple comparisons) vs. saline, METH (1 mg/kg) or METH (2 mg/kg) values. (**E**) SBP values monitored at 3–5 days of withdrawal in mice previously undergoing repeated daily treatments either with saline or METH. Values are reported as the means +/− S.E.M. * *p* < 0.05 (one-way ANOVA for repeated measures with Tukey’s test for multiple comparisons) vs. basal (before treatment) values.

Sensitization to methamphetamine was induced in mice using a protocol consisting of daily administration for 5 consecutive days, as previously reported [32,33,34]. Mice were injected intraperitoneally (i.p.) with saline or METH (1, 2 or 5 mg/kg) daily for 5 consecutive days (day 1–day 5). After 6 days of withdrawal (day 11), mice were re-challenged with either saline or METH. The systolic blood pressure (SBP) was measured by a tail-cuff system 30 min after each administration of saline/METH or after 3–5 days of withdrawal (day 8–day 9) (Figure 1A). This protocol corresponds to our previous studies and studies by other groups aiming to produce METH sensitization. According to this protocol, results are comparable to those obtained following a longer sensitization period, as used by other groups [35,36,37,38]. All mice were killed on day 11 immediately after SBP monitoring, which was performed 30 min after the last saline/METH injection. Dissected brains were used for immunohistochemical analysis of the catecholamine marker tyrosine hydroxylase (TH) and the immediate early gene *c-fos* as a marker of neuronal activation [39] in the nucleus accumbens (NAC), dorsal striatum (STR), substantia nigra pars compacta (SNC), ventral tegmental area (VTA), locus coeruleus (LC) and rostral ventrolateral medulla (RVLM, including the A1/C1 region) (see above).

**Figure 2:**
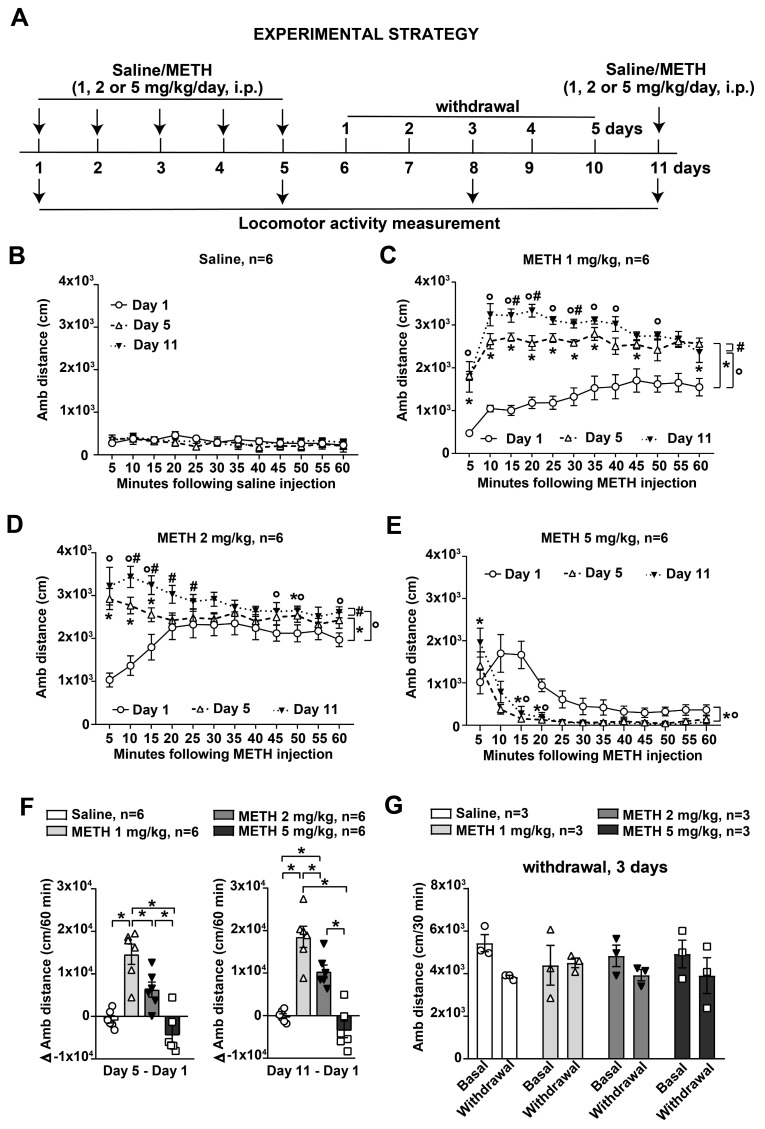
Reiterated exposure to methamphetamine (METH) induces ambulatory sensitization in mice at different doses than SBP sensitization. (**A**) Diagram showing the experimental strategy used for monitoring ambulatory activity in mice subjected to repeated exposure to saline or METH. Mice were daily intraperitoneally (i.p.) injected with saline or methamphetamine (METH, 1, 2 or 5 mg/kg) for 5 days and then they were re-challenged with saline or METH following 6 days of withdrawal at day 11. Ambulatory activity was recorded by an open-field apparatus following injection with saline or METH on day 1, day 5, after the challenge on day 11 and 3 days of withdrawal. Ambulatory activity expressed as ambulatory (Amb) distance traveled for 60 min at 5 min intervals in response to saline or METH at the dose 1 mg/kg, 2 mg/kg and 5 mg/kg on day 1, day 5 and after challenge on day 11 is shown in (**B**), (**C**), (**D**) and (**E**), respectively. Values are expressed as the means +/− S.E.M. Statistical analysis was performed by a 3 × 12 mixed (days vs. minutes) two-way ANOVA for repeated measures with the Tukey’s test for multiple comparisons: * *p* < 0.05 for day 1 vs. day 5. ° *p* < 0.05 for day 1 vs. day 11. # *p* < 0.05 for day 5 vs. day 11. The difference in ambulatory activity (Δ Amb distance) recorded for 60 min following saline or METH injections between days 5 and 1 or days 11 and 1 is shown in (**F**). Values are the means +/− S.E.M. Statistical analysis was performed by one-way ANOVA with Tukey’s test for multiple comparisons: * *p* < 0.05. (**G**) Ambulatory (Amb) distance monitored at 3 days of withdrawal (day 8) following saline or METH (1, 2 or 5 mg/kg, i.p.) repeated injections.

Separate groups of mice subjected to the same experimental paradigm of saline/METH administration were selectively used for the assessment of locomotor activity by an open-field apparatus (see below). Ambulatory activity was assessed for 60 min after saline/METH injection on day 1 (first injection), day 5 (fifth injection) or day 11 (challenge at 6 days of withdrawal) (Figure 2A).

### 2.4. Tail-Cuff Plethysmography

SBP was measured in mice by tail-cuff plethysmography, as previously described [40,41]. Briefly, animals were placed in a holder on a temperature-controlled platform (kept at 37 °C), and recordings were performed in steady-state conditions. SBP values were averaged from at least 3 consecutive measurements.

### 2.5. Open Field Test

Locomotor activity was monitored in an open-field apparatus using boxes (42 × 42 × 21 cm) in association with an activity monitor equipped with an infrared photobeam interruption sensor (Open Field Activity System Hardware, Med Associates, Inc., St. Albans, VT, USA). Mice were individually placed into the test box in a quiet room for 60 min prior to injection to allow locomotor activity to stabilize (habituation). Then, mice were injected with saline or METH and immediately placed into the same test box to record locomotor activity for 60 min with 5 min intervals.

### 2.6. Immunohistochemistry

**Figure 3:**
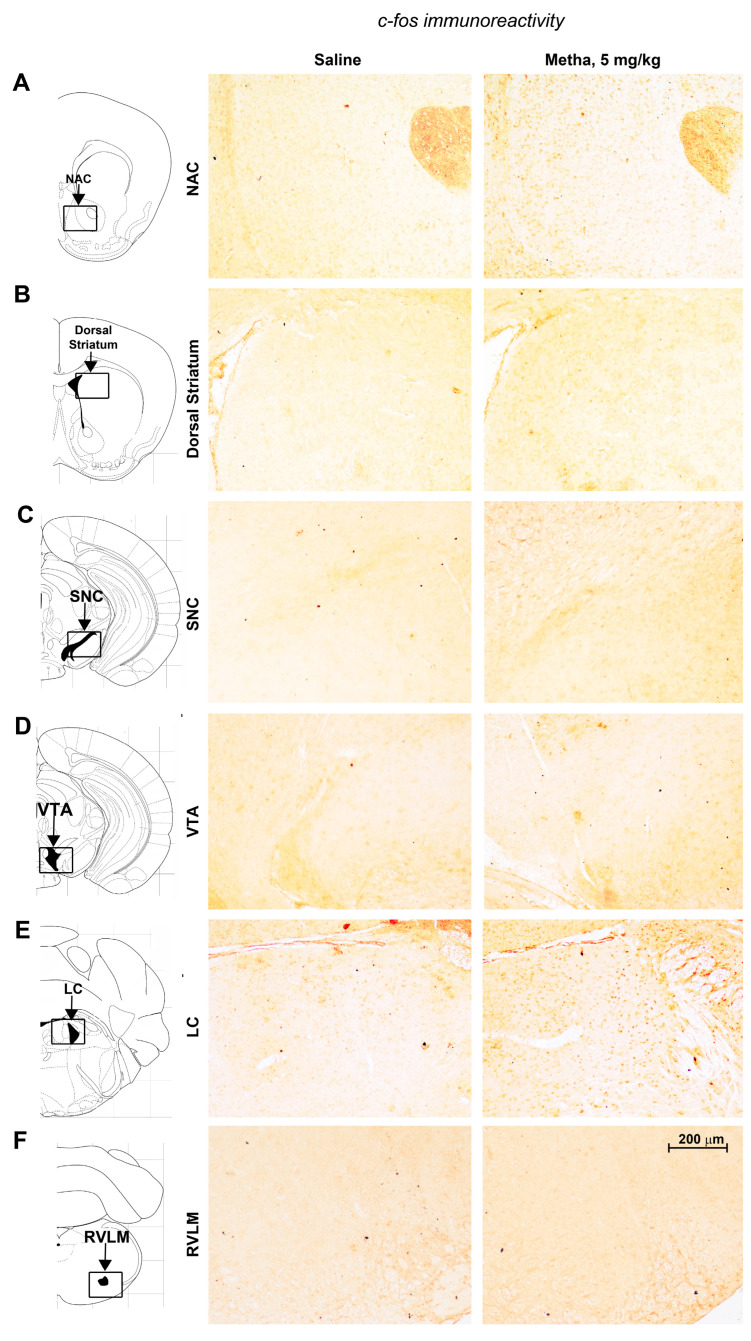
Regional pattern of neuronal activation induced in response to reiterated exposure to METH. Immunohistochemical analysis of the c-fos was performed in dissected brains of mice subjected to repeated exposure to saline or METH (5 mg/kg, i.p.) and killed 60 min after the challenge at day 11. Representative images of c-fos-positive cells in the nucleus accumbens (NAC) (**A**), dorsal striatum (**B**), substantia nigra pars compacta (SNC) (**C**), ventral tegmental area (VTA) (**D**), locus coeruleus (LC) (**E**) or rostral ventrolateral medulla (RVLM) (**F**) are shown in the panel.

Dissected brains were fixed in Carnoy’s solution (60% ethanol, 10% acetic acid, 30% chloroform) and embedded in paraffin. Fifteen serial sections of NAC, STR, SNC, VTA, LC and RVLM were cut by a microtome (Leica RM2245, Wetzlar, Germany) and used for immunohistochemical analysis of TH or c-fos immunoreactivity (Figure 3). Sections were deparaffinized by immersion in xylene (30 min) and rehydrated by serial dilutions of ethanol. For antigen retrieval, sections were treated with a 10 mM, pH 6.0 citrate buffer heated in a microwave for 20 min. After that, sections were treated at RT for 15 min with 0.1% triton X-100 for permeabilization and then incubated for 10 min at room temperature (RT) in 3% hydrogen peroxide to eliminate endogenous peroxidase activity. Blocking was performed by incubation for 60 min at RT in phosphate buffer solution (PBS)/6% normal horse serum (NHS) for TH or PBS/6% normal rabbit serum (NRS) for c-fos. Afterward, slices were incubated overnight at 4 °C with the primary antibody mouse monoclonal anti-TH (Sigma Aldrich, code #T1299, 1:100 in PBS/3% NHS) or sheep polyclonal anti-c-fos (EMD Millipore, Temecula, CA, USA, 1:100, code #AB1584, 1:100 in PBS/3% NRS) and then with a secondary biotinylated anti-mouse (1:400 in PBS, 10 min at RT; Vector Laboratories, Burlingame, CA, USA, code #BA-2000) or anti-sheep (1:200 in PBS, 60 min at RT; Vector Laboratories, code #BA-6000) antibody. Finally, slices were incubated for 5 min at RT with horseradish peroxidase-conjugated streptavidin (1:100 in PBS; Vector Laboratories, code #SA-5004) and then for 3 min with 3,3-diaminobenzidine tetrachloride (Sigma Aldrich, code: #D4293-50set). Immunoreactivity was analyzed by using a bright field microscope. Images of the regions of interest were digitally collected and counted with ImageJ software (version number 6.2.1.491). TH-positive cell density was assessed within dissectors of known dimension (900 μm^2^) randomly positioned by the software Image Pro Plus 6.2 within the area of interest (AOI) drawn by the operator at low magnification (2.5×). Inside each dissector, the TH-positive cells were detected and counted at high magnification (100×). Cell density was expressed as a number per mm^2^.

### 2.7. Statistical Analysis

**Figure 4:**
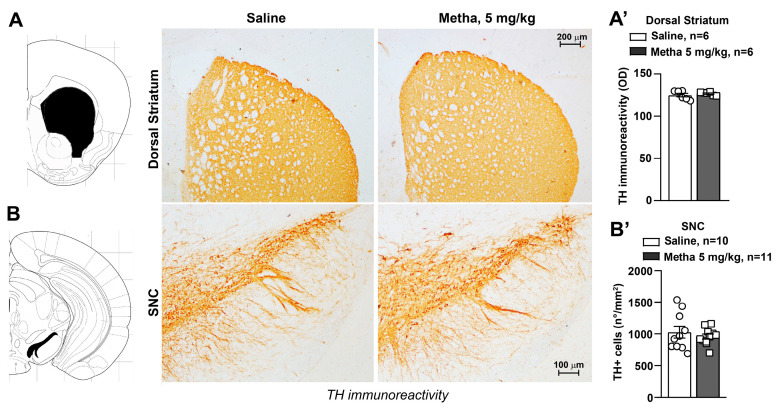
Repeated administrations of METH do not damage the catecholamine nigrostriatal system. Mice were daily injected i.p. either with saline or methamphetamine (METH, 5 mg/kg) for 5 days and then, re-challenged with saline or METH following 6 days of withdrawal at day 11. Immunohistochemical analysis of the catecholamine marker TH was performed in dissected brains of mice killed at 60 min after the challenge. Representative images of TH-positive fibers in the dorsal striatum and TH-positive cells in the substantia nigra pars compacta (SNC) are shown in (**A**) and (**B**), respectively. Graphs in (**A’**) and (**B’**) show densitometric analysis of striatal TH immunoreactivity (OD: optical density) and TH-positive cell density (n°/mm^2^), respectively. Values are the means + S.E.M.

**Figure 5:**
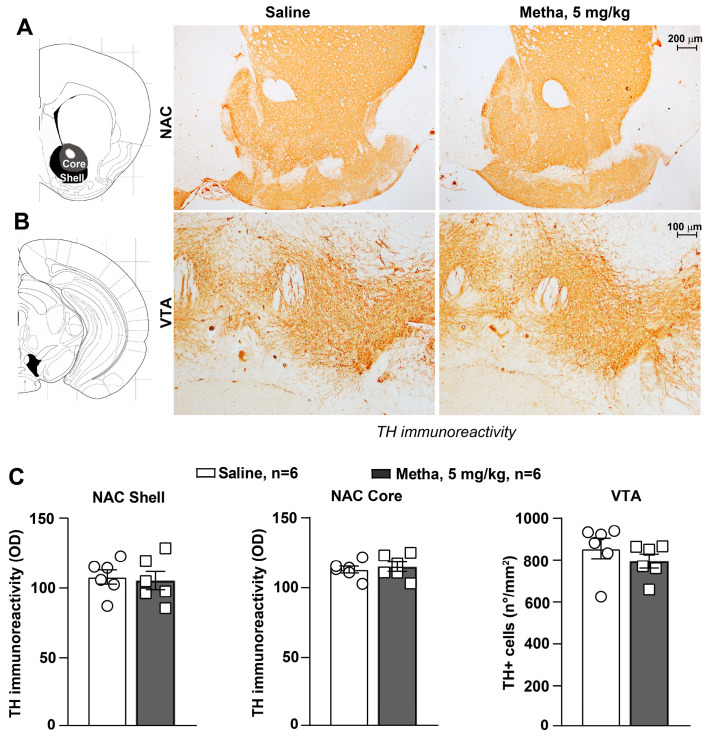
Reiterated exposure to METH did not damage the catecholamine meso-accumbal system. Immunohistochemical analysis of the catecholamine marker tyrosine hydroxylase (TH) was carried out in dissected brains of mice repeatedly injected either with saline or METH (5 mg/kg, i.p.) and killed 60 min after the challenge on day 11. Representative images of TH-positive fibers in the nucleus accumbens (NAC) and TH-positive cells in the ventral tegmental area (VTA) are shown in (**A**) and (**B**), respectively. Graphs in (**C**) show densitometric analysis of striatal TH immunoreactivity (OD: optical density) in the shell and core of the NAC and TH-positive cell density (n°/mm^2^) in the VTA. Values are the means + S.E.M.

**Figure 6:**
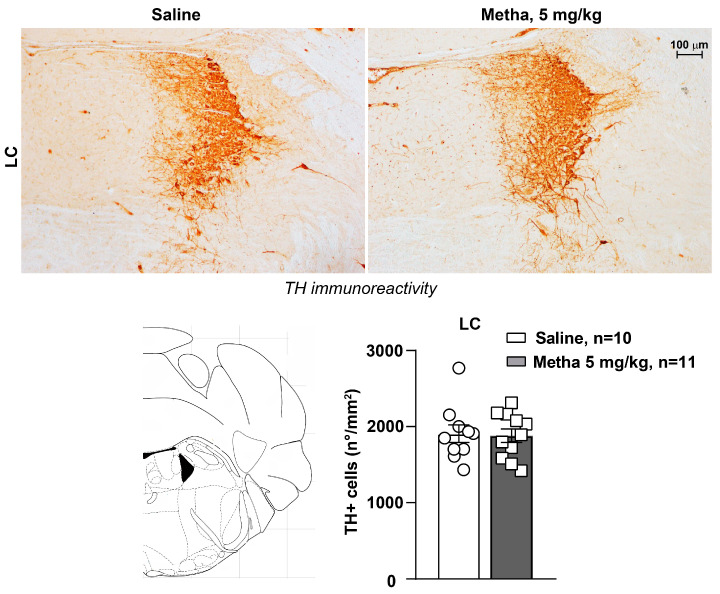
Repeated exposure to METH does not alter noradrenergic neurons in the locus coeruleus (LC). Immunohistochemical analysis of the catecholamine marker tyrosine hydroxylase (TH) was performed in dissected brains of mice subjected to repeated exposure to saline or METH (5 mg/kg, i.p.) and killed 60 min after the challenge on day 11. Representative images of TH-positive neurons in the locus coeruleus (LC) of mice are shown in the panel. The graph shows values of TH-positive cell density (n°/mm^2^) in the LC. Values are the means + S.E.M.

Statistical analysis was performed by using the GraphPad Prism Software, version 8.0.1, as follows: (i) one-way ANOVA for repeated measures with Tukey’s test for multiple comparisons (Figure 1B,E); (ii) one-way ANOVA with Tukey’s test for multiple comparisons (Figure 1C,D and Figure 2F); (iii) two-way ANOVA for repeated measures with Tukey’s test for multiple comparisons (Figure 2B–E,G); (iv) unpaired two-tailed Student *t*-test (Figure 4, Figure 5 and Figure 6). Data are shown as mean ± standard error of the mean (S.E.M.). *p*-values < 0.05 were considered statistically significant.

## 3. Results

### 3.1. Repeated Injection with the Highest Dose of METH Induced SBP Sensitization in Mice

SBP was monitored using the tail-cuff method 30 min after each injection or during the phase of withdrawal in mice subjected to reiterated injections of saline or METH (1, 2 or 5 mg/kg, i.p.) (Figure 1A).

No changes in SBP values were observed in response to the first injection with saline or METH on the first day with respect to basal levels (recorded before treatment) (Figure 1B), while a gradual increase in SBP was detected in the first 3 days of daily injection in response to the higher dose of METH (5 mg/kg). Following the fourth and fifth METH administrations at the same dose, no further increase in SBP was detected (Figure 1B). SBP values were not affected in response to treatment with saline (Figure 1B, F_2.328,11.64_ = 0.2160, *p* > 0.05). Only a negligible increase (6%) in SBP was detected following the last METH treatments at the lower doses (1 or 2 mg/kg, i.p.) (Figure 1B) (METH 1 mg/kg: F_2.279,9.117_ = 4.266, *p* < 0.05; METH 2 mg/kg: F_1.538,6.151_ = 1.432, *p* > 0.05; METH 5 mg/kg: F_2.313,11.56_ = 20.18, *p* < 0.05).

All mice were re-challenged with saline or METH (1, 2 or 5 mg/kg, i.p.) after 6 days of withdrawal (i.e., on day 11) and SBP values were monitored 30 min after the treatment. No changes in SBP values were observed in response to a challenge with saline or the lower doses of METH (1 or 2 mg/kg, i.p.) (Figure 1C). On the contrary, treatment with the highest dose of METH (5 mg/kg, i.p.) following 6 days of withdrawal after the last dose of the repeated exposure protocol still led to increased SBP compared to values recorded in control saline- or METH-injected (1 or 2 mg/kg, i.p.) mice (Figure 1C, F_3,18_ = 8.603, *p* < 0.05). Then, repeated exposure to METH induced a hypertensive response that was maintained despite the interruption in daily treatments, suggesting the occurrence of sensitization by persistent plastic changes in molecular mechanisms underlying SBP control.

The extent of METH-induced SBP sensitization was calculated as differences in SBP values (δ) monitored following the fifth treatment (day 5) or after the challenge (day 11) and the respective SBP values recorded in response to the first treatment on day 1 (Figure 1D). No changes in Δ-SBP values were detected in response to treatments with the lower doses of METH (1 or 2 mg/kg) with respect to saline injections (Figure 1D). Remarkably, data showed an increase in Δ-SBP values in response to the highest dose of METH (5 mg/kg) compared to saline or METH (1 or 2 mg/kg, i.p.) injection both for day 5 vs. day 1 and day 11 vs. day 1, mirroring the occurrence and long-term maintenance of SBP sensitization, respectively (Figure 1D) (Day 5–Day 1: F_3,18_ = 20.04, *p* < 0.05; Day 11–Day 1: F_3,18_ = 7.09, *p* < 0.05).

Measurements of SBP during the withdrawal showed increased SBP values in mice subjected to repeated daily treatment with the higher dose of METH (5 mg/kg) after 5 days of withdrawal with respect to basal levels (recorded before treatments) (Figure 1E). These data suggest the long-term persistence of plastic changes at the level of SBP control mechanisms in response to reiterated exposure to METH (Figure 1E, saline: F_2.022,10.11_ = 0.1783, *p* > 0.05; METH 5 mg/kg: F_1.754,8.771_ = 3.477, *p* > 0.05).

### 3.2. Ambulatory Sensitization in Response to METH

Ambulatory activity was monitored in an open-field apparatus. Mice were treated with saline or METH (1, 2 or 5 mg/kg, i.p.) daily for 5 days and re-challenged with either saline or METH after 6 days of withdrawal (i.e., on day 11). The ambulatory distance was assessed for 60 min at 5 min intervals on days 1, 5 and 11 (Figure 2A). There was no difference between ambulatory distance monitored on day 1 (following the first injection), day 5 (following the fifth repeated injection) and day 11 (following the challenge) in saline-treated mice (Figure 2B, minutes: F_3.069,15.35_ = 1.849, *p* > 0.05; days: F_1.223,6.116_ = 0.6267, *p* > 0.05; minutes × days: F_4.01,20.05_ = 1.026, *p* > 0.05). Locomotor sensitization was detected in mice treated with the lower doses of METH (1 or 2 mg/kg), with responses being progressively greater on day 5 and following METH challenge on day 11 (Figure 2C, minutes: F_2.5,12.5_ = 12.67, *p* < 0.05; days: F_1.232,6.160_ = 44.65, *p* < 0.05; minutes × days: F_2.848,14.24_ = 3.435, *p* < 0.05; Figure 2D, minutes: F_1.918,9.590_ = 0.9982, *p* > 0.05; days: F_1.307,6.537_ = 27.10, *p* < 0.05; minutes × days: F_2.719,13.60_ = 9.312, *p* < 0.05).

Paradoxically, locomotor activity was progressively decreased in response to the highest METH dose (5 mg/kg) (Figure 2E, minutes: F_1.497,7.485_ = 23.34, *p* < 0.05; days: F_1.093,5.466_ = 25.58, *p* < 0.05; minutes × days: F_1.602,8.008_ = 4.418, *p* > 0.05). This suggests an ambulatory desensitization for daily doses of 5 mg/kg, in line with some previous data [33].

The extent of METH-induced ambulatory sensitization was compared by assessing the Δ increase in ambulatory distance between values monitored following the fifth treatment (day 5) or after the challenge (day 11) and the respective ambulatory distance values recorded in response to the first treatment on day 1 (Figure 2F, Day 5–Day 1: F_3,20_ = 20.47, *p* < 0.05; Day 11–Day 1: F_3,20_ = 31.20, *p* < 0.05). Data showed a significant Δ increase in ambulatory distance between values monitored following the fifth treatment (day 5) and the respective ambulatory distance values recorded in response to the first treatment on day 1, only occurring in response to daily treatment with the lowest dose of METH (1 mg/kg, i.p.) compared to saline-injected mice (Figure 2F). This suggests that the highest ambulatory sensitization was induced in mice in response to repeated administration of METH at the dose of 1 mg/kg. The dose of 2 mg/kg was still able to induce ambulatory sensitization, although to a lesser extent compared with the dose of 1 mg/kg (Figure 2F).

Ambulatory activity was monitored after 3 days of withdrawal. In contrast to that observed for values of SBP, during METH withdrawal, ambulatory-sensitized mice did not show an increase in locomotor activity compared with basal values (before treatment) (Figure 2G, treatment: F_3,8_ = 0.0536, *p* > 0.05; day: F_1,8_ = 9.971, *p* < 0.05; treatment × day: F_3,8_ = 1.727, *p* > 0.05).

### 3.3. METH Treatment Led to a Specific Regional Profile of c-fos-Related Neuronal Activation

We examined the expression of the immediate early gene *c-fos* as a marker of neuronal activation [39] in the NAC, STR, SNC, VTA, LC and RVLM of mice treated daily with saline or METH (5 mg/kg, i.p.) for 5 consecutive days and re-challenged with saline or METH following 6 days of withdrawal (day 11). The immunohistochemical analysis was performed on dissected brains of mice killed on day 11 immediately after the SBP monitoring performed 30 min after the last saline/METH injection. Data showed a specific pattern of regional activation in response to repeated exposure to METH. In detail, we detected a robust increase in c-fos positive cells in the NAC (Figure 3A) and LC (Figure 3E), while no changes in c-fos positive cells were detected either in the STR (Figure 3B), SNC (Figure 3C), VTA (Figure 3D) or RVLM (Figure 3F). Therefore, repeated exposure to METH led to neuronal activation in specific brain regions, such as the LC, but not A1/C1 within the RVLM, which represent key areas in modulating blood pressure.

### 3.4. METH Treatment Did Not Induce Catecholamine Neuron Damage

Immunohistochemical analysis was performed to evaluate the occurrence of morphological alterations within nigrostriatal, meso-limbic and pontine catecholaminergic systems in the brains of mice treated with METH compared with saline-treated control mice. The analysis of TH immunoreactivity showed that the repeated daily administration of METH, at the dose of 5 mg/kg, did not induce changes in striatal DA innervation (Figure 4A, *p* > 0.05) or a loss in SNC neurons (Figure 4B, *p* > 0.05).

Similar results were obtained for the meso-accumbal pathway, where no changes in catecholamine innervation were detected either in the shell or in the core of the NAC or in TH-positive neuronal density within the VTA in response to METH daily treatments (Figure 5, *p* > 0.05).

Similarly, we did not detect any alteration in response to repeated treatments with METH (5 mg/kg) concerning TH-positive cell density within the pontine nucleus LC (Figure 6, *p* > 0.05).

## 4. Discussion

The present manuscript indicates that repeated exposure to METH produced in mice a persistent elevation in SBP, following a sensitization process consisting of repeated daily administrations of METH for 5 days. Indeed, after 3 days, SBP sensitization was already achieved since additional administrations on day 4 and day 5 did not further increase SBP. These results strongly depend on the dose of METH being administered since lower doses (1 mg/kg and 2 mg/kg) did not produce SBP sensitization, even when measured at 5 days. The sensitization process was challenged 11 days following the beginning of the experimental protocol. At this time, the increase in SBP was enhanced compared with basal conditions before treatment (naïve mice). Still, the increase in SBP induced by METH when observed on day 11 was slighter compared with day 3 during the sensitization process. It is remarkable that, following sensitization, during METH withdrawal, SBP remained elevated compared with baseline conditions. This phenomenon indicates a persistent up-regulation of SBP that is triggered by previous METH exposure, although it persists independently of METH administration and adds to the sensitization process. In fact, the sensitization process was slighter from day 3 up to day 5, when the measurement of SBP was likely to be the consequence of a persistent SBP up-regulation and the sensitization process was vanishing. In fact, during the sensitization process, the increase in SBP measured at day 4 and day 5 following METH was reduced compared with SBP measured at day 3, which was significantly increased compared with day 2 and day 1 and baseline levels. These data suggest that daily administration of METH at 5 mg/kg requires just 3 days to produce the following phenomena: (i) persistent elevation in blood pressure independently of further METH administrations and (ii) exaggerated response to administration of the same dose of METH even at 6 days withdrawal. When these data are confirmed in human beings, this will be relevant for METH abuse and cardiovascular risk since a few doses of METH are enough to enhance the increase in SBP induced by further METH exposure, which leads to unpredictable risks of cardiovascular emergencies, even for occasional, non-addicted abusers. Most importantly, a few METH administrations may produce a persistent alteration that may last an unpredictable amount of time to produce a condition of systolic hypertension. It is remarkable that the occurrence of plastic changes in SBP regulation follows a dose–response curve that differs from that occurring for METH-induced locomotor sensitization. In fact, when mice were administered METH at 1 mg/kg, marked locomotor sensitization was observed, which was evident with the challenging dose on day 11. The dose of 2 mg/kg was still able to induce ambulatory sensitization, although this was significantly slighter compared with sensitization induced by METH at a dose of 1 mg/kg. The dose of 5 mg/kg, which sensitized SBP, was negligible concerning ambulatory sensitization as much as 1 mg/kg or 2 mg/kg METH doses were non-effective in producing sensitization of SBP. The other seminal difference concerning ambulatory compared with SBP sensitization was the persistency of elevated SBP levels, even in the absence of a challenge. In fact, mice sensitized with 5 mg/kg METH maintained higher SBP during the withdrawal interval. In contrast, ambulatory-sensitized mice moved just as slowly as naïve mice during withdrawal. This dose-dependent difference and the alterations during the withdrawal window suggest the occurrence of two specific and different biological mechanisms underlying these ambulatory and SBP sensitizations. The brain areas analyzed for morphology did not allow us to establish a difference compared with saline-injected control mice, even concerning the effects induced by METH (5 mg/kg). In fact, repeated daily METH administration at the dose of 5 mg/kg was neither reported to produce damage nor an increase in DA innervation to nigrostriatal and meso-accumbal pathways. This is in line with the current literature, where METH needs to be administered for at least 21 days at a dose of 5 mg/kg to reduce nigrostriatal DA innervation and alter ventral meso-striatal TH immunostaining [42]. Similarly, we did not detect any alteration concerning the cell number within the pontine nucleus LC, which is a target of METH and represents a key area in modulating blood pressure. Indeed, despite no different cell numbers being measured in the LC, we detected a robust increase in the number of c-fos positive cells in the LC in response to repeated exposure to METH (5 mg/kg). These data demonstrate a selective regional profile of neuronal activation mirroring specific patterns of events that can be responsible for alterations in cardiovascular homeostasis. In contrast, no change in the morphology of the A1/C1 area was detected when c-fos immunostaining was carried out.

Moreover, cell shape and mostly cell volume were apparently reduced following sensitization, which requires an analytical study of LC neurons at the subcellular level. Such a study, which is in progress, should feature all the relevant intracellular targets of METH within catecholamine cells. The doses producing SBP sensitization were below the threshold for inducing neurotoxicity. In fact, the loss of DA axons arising from SNC was produced at the dose of 5 mg/kg METH starting at 14 days of daily METH administration. Similarly, the loss of NE axons arising from the LC was detected as early as day 14 following the administration of 5 mg/kg METH. Both nuclei were affected concerning a decrease in neuron numbers, only following a double cumulative dose of METH (28 days of METH at 5 mg/kg) [28]. In this study, a stereological approach was used and the effects on axonal integrity were assessed by stereology of axonal length. The biological mechanisms and site-specificity for METH-induced sensitization to blood pressure remain to be determined. The present study provides novel evidence compared with previous phenomena produced by METH-induced sensitization. Although, LC neurons do not seem to be affected by the occurrence of SBP sensitization and RVLM does not seem to be activated. Other nuclei belonging to the brainstem reticular formation should be investigated concerning changes in the amount of catecholamine cells, oxidative stress, and neurodegeneration. Further analysis could involve preganglionic sympathetic nuclei of the spinal cord as well as the sympathetic ganglia, the carotid body and sinus, as well as the amount of noradrenergic innervation of the heart and main blood vessels. Again, further works should be dedicated to the study of arterial intrinsic vascular reactivity, the functional anatomy and biochemistry of the kidney, and the role of the spleen in blood pressure. Innumerous sites possess significant NE innervation, which is a potential target of METH, which is taken up by NE axons. Therefore, just like the stream of literature about behavioral and locomotor sensitization induced by psychostimulants, which is composed of thousands of papers published during the last few decades, it will take significant efforts to dissect which sites and molecular mechanisms govern the onset and maintenance of the METH-induced plasticity of blood pressure.

## 5. Conclusions

The present study indicates that repeated METH exposure, daily, for 5 days, produces in mice an increase in SBP which undergoes sensitization. Similarly, such a daily administration protocol produces a sensitization of locomotor activity. It is remarkable that SBP sensitization leads additionally to persistent self-sustaining SBP elevation. This occurs with a dosing of METH that differs from that required for ambulatory sensitization. As far as cell density is considered, none of these effects appears to be associated with morphological changes within SNC, VTA or LC. Instead, the morphology of c-fos as an index of neuronal activity appears to increase in the LC but not in the RVLM (including the A1/C1 region).

These data suggest a need to re-consider the motor and addictive effects of METH, which are similar to cardiovascular effects, concerning the ability to produce plastic phenomena that enhance the effects following repeated exposure (sensitization). Despite being similar, these phenomena are likely to depend on different morphological/biochemical substrates since the doses of METH diverge. The specific site that may be responsible for these effects remains to be established. Similarly, the mechanisms that may sustain such an effect need to be investigated.

## Data Availability

The data for the current study are available from the corresponding author upon reasonable request.
